# Effect of Heat Stress on Sperm DNA: Protamine Assessment in Ram Spermatozoa and Testicle

**DOI:** 10.1155/2018/5413056

**Published:** 2018-03-25

**Authors:** Thais Rose dos Santos Hamilton, Adriano Felipe Perez Siqueira, Letícia Signori de Castro, Camilla Mota Mendes, Juliana de Carvalho Delgado, Patrícia Monken de Assis, Leonardo Pereira Mesquita, Paulo César Maiorka, Marcílio Nichi, Marcelo Demarchi Goissis, José Antonio Visintin, Mayra Elena Ortiz D. Ávila Assumpção

**Affiliations:** ^1^Department of Animal Reproduction, School of Veterinary Medicine and Animal Science, University of Sao Paulo, Av. Prof. Dr. Orlando Marques de Paiva, 87, 05508 270, Cidade Universitária, Sao Paulo, SP, Brazil; ^2^Department of Animal Pathology, School of Veterinary Medicine and Animal Science, University of Sao Paulo, Av. Prof. Dr. Orlando Marques de Paiva, 87, 05508 270, Cidade Universitária, Sao Paulo, SP, Brazil

## Abstract

Sperm DNA fragmentation is considered one of the main causes of male infertility. The most accepted causes of sperm DNA damage are deleterious actions of reactive oxygen species (ROS), defects in protamination, and apoptosis. Ram sperm are highly prone to those damages due to the high susceptibility to ROS and to oxidative stress caused by heat stress. We aimed to evaluate the effects of heat stress on the chromatin of ejaculated and epididymal sperm and the activation of apoptotic pathways in different cell types in ram testis. We observed higher percentages of ejaculated sperm with increased chromatin fragmentation in the heat stress group; a fact that was unexpectedly not observed in epididymal sperm. Heat stress group presented a higher percentage of spermatozoa with DNA fragmentation and increased number of mRNA copies of transitional protein 1. Epididymal sperm presented greater gene expression of protamine 1 on the 30th day of the spermatic cycle; however, no differences in protamine protein levels were observed in ejaculated sperm and testis. Localization of proapoptotic protein BAX or BCL2 in testis was not different. In conclusion, testicular heat stress increases ram sperm DNA fragmentation without changes in protamination and apoptotic patterns.

## 1. Introduction

Infertility, poor embryonic development, and genetic abnormalities in the offspring have been linked to sperm DNA damage [1, 2]. This occurs because sperm with defective chromatin are still capable to fertilize oocytes [3]. Mouse oocytes fertilized with sperm carrying DNA damage caused adverse effects on embryonic development, postnatal growth, litter longevity, and tumor susceptibility [4]. However, the exact causes that lead to the abnormalities in chromatin and sperm DNA damage are not precisely known. The three most accepted causes are [3, 5] oxidative stress, defective packaging of sperm chromatin, and apoptosis.

Oxidative stress directly or indirectly causes sperm DNA fragmentation. An imbalance between the production of reactive oxygen species (ROS) and activity of antioxidants triggers DNA damage. Such imbalance generates base damage and breaks in the double stranded DNA, inducing sperm DNA fragmentation [6]. Some studies found a clear relationship between heat and oxidative stresses [7, 8]. Other studies in rams have reported a reduction of sperm DNA integrity under heat stress conditions induced by testicular insulation [9, 10]. Oxidative stress was described as the main cause of sperm DNA fragmentation [11] and may be related to protamine deficiency and apoptosis.

In mammalian spermatogenesis, transition proteins replace the majority of histones in spermatids destabilizing nucleosomes, preventing DNA torsion, facilitating the DNA repair of strand breaks, and contributing to chromatin condensation [12–14]. Subsequently, positively charged proteins called protamines replace these transition proteins, in a process termed protamination [15, 16]. When protamination occurs incorrectly, disorders in sperm chromatin condensation can appear [17], with implications on fertility [18]. Sperm chromatin packaging not only causes compaction of the genetic material but also protects the sperm DNA of physical and chemical damage [12–14]. Protamine deficiency makes spermatozoa more vulnerable to the action of free radicals [17]; thus, it is possible that heat stress may be influencing protamination process as abnormal sperm chromatin was described under heat stress conditions [19–22]. In fact, sperm lipid peroxidation causes changes in expression of genes related to the protamination process [10].

Sperm DNA fragmentation caused by heat stress and consequent oxidative stress can lead to apoptosis. Apoptosis often occurs in testicular germ cells [23] and is associated with sperm maturation [24]. However, factors such as heat, toxic agents, and radiation increase apoptotic rates [25], and some researchers have reported cell damage resulting from apoptosis after complete spermatogenesis [26, 27]. In this context, damages generated by ROS may be associated with apoptosis and can be detected by specific markers [5]. BAX and BCL2 proteins are examples of apoptotic signaling molecules. An increase in the levels of BAX and/or a decrease in BCL2 levels are known to promote permeabilization of the mitochondrial membrane leading to the release of proapoptotic factors [28].

It is believed that the heat stress causes sperm DNA modifications in ram sperm [29]. In this study, we address the influence of heat stress on the three most accepted causes of sperm chromatin abnormalities: oxidative stress, defects in protamination, and apoptosis. Little is known about the interference of heat stress on sperm DNA compaction in rams. A few data considering the expression of replacement proteins or protamination failures are available. Moreover, it is not known if there is activation of apoptotic routes mediated by ROS in ram testis. In order to clarify these issues, we evaluated sperm DNA damage, chromatin structure, and protamination-related proteins in ram ejaculated sperm over 9 weeks after heat stress induction by testicular insulation and in ram epididymal sperm immediately and 30 days after testicular insulation. We also evaluated protamine and transition protein (TNP1 and TNP2) gene expression in ejaculated and epididymal sperm and in testicular tissue of ram subjected to heat stress by testicular insulation. Furthermore, we analyzed the immunolocalization of protamines and pro- and antiapoptotic proteins BAX and BCL2 in ram testicular tissue.

## 2. Material and Methods

Unless otherwise indicated, all chemicals were obtained from Sigma Chemicals (St. Louis, MO). The experiments were performed using twelve mature (8 months old) Santa Ines rams. Animals belonged to the Department of Animal Reproduction of the School of Veterinary Medicine and Animal Science from the University of Sao Paulo. Animals were submitted to uniform nutritional conditions, and the experiments were approved by the Bioethics Committee of the School of Veterinary Medicine and Animal Sciences, University of Sao Paulo (protocol number 2445-2011).

### 2.1. Experiment 1: Effects of Heat Stress on Ejaculated Sperm DNA

**Figure 1:**
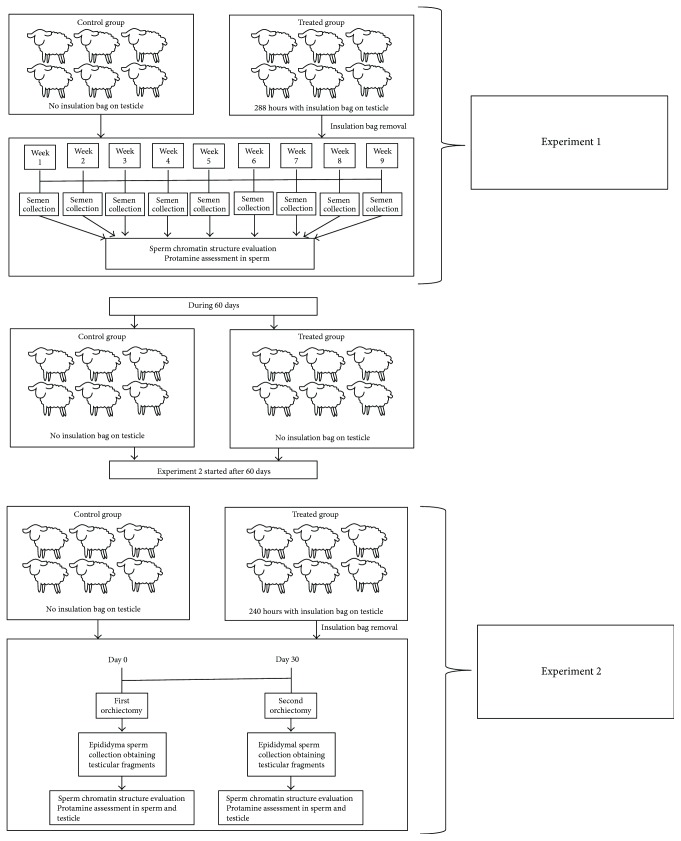
Experimental design flow chart.

The experimental design was similar to our previous published study [29]. Animals were randomly divided in two groups, control (*n* = 6) and treated (*n* = 6). In the heat stress (treated) group, animals underwent testicular insulation using an insulating bag placed in the scrotum of animals, in order to induce heat stress effects on spermiogenesis. The bags were kept for 288 consecutive hours, and during this period, the internal temperature of each bag and environmental temperature were monitored using a digital thermometer. After removal of the bags (week 1), fresh ram semen was collected weekly using an artificial vagina during nine weeks. Experimental design flow chart is presented in Figure 1.

#### 2.1.1. Sperm Chromatin Structure Evaluation

*(1) Assessment of Sperm DNA Susceptibility to Denaturation*. Sperm DNA fragmentation was evaluated by flow cytometry using Guava Easycyte Mini System (Guava Technologies, Hayward, CA, USA) after acid induced denaturation using acridine orange as previously described [10]. Red color indicates the presence of single-stranded DNA, which designates denaturation of the chromatin structure, while green color indicates the presence of double-stranded DNA. The same protocol was performed in experiment 2 for epididymal sperm.

*(2) Assessment of DNA Integrity by a Modified Alkaline Comet Assay*. To assess sperm DNA integrity, we used the modified alkaline comet assay as previously described [10]. The image display was obtained using an Olympus IX epifluorescence microscope equipped with an Olympus Q-Color™ camera (Olympus, Tokyo, Japan), using 515–560 nm excitation filters and 600 nm barrier filters. The images of 200 cells were randomly selected and analyzed by visual grading in grades I to IV according to the intensity of DNA damage, ranging from little DNA fragmentation to intense DNA fragmentation, as previously described [10]. Briefly, grade I indicates little DNA damage, grade II indicates average DNA damage, grade III indicates obvious DNA damage, and grade IV indicates intense DNA damage.

#### 2.1.2. Protamine Assessment in Sperm

Protamine characterization was assessed by protamine deficiency (CMA3) in sperm chromatin, gene expression of protamine 1 and transition proteins (1 and 2) in spermatozoa, and quantification of protamine (1 and 2) in isolated sperm nuclei.

*(1) Protamine Deficiency in Sperm Chromatin*. We used chromomycin A3 (CMA3) fluorochrome to investigate protamine deficiency in ram sperm by flow cytometry as previously described [10]. About 30 million spermatozoa from each ejaculate were washed in PBS without calcium (Ca) and magnesium (Mg) by centrifugation at 9000*g* for 60 seconds. Samples were fixed in Carnoy's solution (3 parts methanol : 1 part of acetic acid) at 4°C for 10 minutes followed by a wash as described above. The pellet was resuspended and stained with 0.25 mg/ml CMA3 solution for 20 minutes at room temperature and in the dark. Samples were then washed as described above. After suspension of the pellet in PBS, samples were submitted to flow cytometry analysis using the 583 nm detector (PM1) of the Guava Easycyte Mini System. Decondensed sperm nucleus and extraction of protamines were used as control, in order to obtain a deprotaminated cell pattern [10].

*(2) Gene Expression of Protamine 1, Transition Proteins 1 and 2 in Ram Ejaculated Sperm*. One sample from every ejaculate, from each animal, was used for RNA isolation (*n* = 108). After semen collection, samples with 10^6^ spermatozoa were washed in PBS. About 500 *μ*L of RNA Later solution (Life Technologies, Carlsbad, USA) was added to the pellet to stabilize RNA. Samples were kept in liquid nitrogen until RNA isolation. Spermatozoa membrane was disrupted (snap freezing, 10 rounds of freezing and thawing in liquid nitrogen), and then the RNA isolation was carried using TRIzol, according to the manufacturer's instructions (Life Technologies, Carlsbad, USA). Traces of DNA were removed by DNAse treatment using the PureLink™ DNAse (Invitrogen, Carlsbad, CA, USA). Isolated RNA was evaluated using NanoDrop ND-1-spectrophotometer (Thermo Scientific), and 280/260 values between 1.9 and 2.1 were considered adequate. Complementary DNA was synthesized using Superscript VILO cDNA Synthesis kit following the manufacturer's instructions (Life Technologies, Carlsbad, USA). Quantification of cDNA was performed using Qubit dsDNA BR and Qubit 2.0 Fluorometer (Life Technologies), and cDNA samples were diluted to 2.5 *μ*g/mL. Primers for protamine 1 (PRM*1*-forward primer: ACAGTAACCGCACAGTAGCA, reverse primer: GTGGCATTGTTCGTCAGCAG), transition protein 1 (TNP1-forward primer: GCTGTGATGATGCCAATCGC, reverse primer: GTCCCCCTTCTGTTCGGTTG), and transition protein 2 (TNP2-forward primer: GTCCCCCTTCTGTTCGGTTG, reverse primer: TCAGTTGTACTTCCGTCCTGAG) were designed using the online software Primer-BLAST (http://www.ncbi.nlm.nih.gov/tools/primer-blast) based on the sequences of the respective *Ovis aries* transcripts (NM001163050.0 protamine 1, XM_004004917.1 predicted transition protein 1, XM_004020771.1 predicted transition protein 2). PCR was first carried out to verify the presence of these transcripts in spermatozoa. Then, absolute quantitative PCR (q-RT-PCR) was performed to calculate the number of copies of target genes in each sample as described previously [10]. SYBR GreenER qPCR Supermix Universal kit was used for qPCR (Life Technologies, Carlsbad, USA) in a Mastercycler ep realplex Thermal Cycler (Eppendorf AG, Hamburg, Germany) (40 amplification cycles of 95°C for 15 seconds and 60°C for 60 seconds). The same procedure was performed in experiment 2, using epididymal sperm.

*(3) Quantification of Protamines 1 and 2 in Ram Sperm*. Sperm nuclei were isolated, as previously described for human sperm [30], with some modifications. This protocol consists in the use of CTAB detergent (cetyltrimethylammonium bromide) for the removal of the spermatozoon tail. Therefore, 20 × 10^6^ spermatozoa were incubated on ice in 50 mMTris-HCl (pH 8) solution and 10 mM DTT, for 15 minutes. After this, CTAB was added to the samples at a final concentration of 0.1%. After 30 minutes of incubation on ice, samples were washed twice in 50 mM Tris-HCl (pH 8) (3000G/5 minutes at 4°C). The pellet containing the cells was stored at −20°C for further analyses. Nuclear sperm proteins were extracted as previously described for human sperm [31] after nuclear isolation with CTAB. This protocol uses 650 mM NaCl to separate proteins from the soluble fraction, which consists of supernatant containing proteins with lower DNA affinity such as histones, and insoluble fraction consisting of a pellet containing proteins with higher DNA affinity, such as protamines. After extracting fractions, proteins were precipitated with 20% trichloroacetic acid (*w*/*v*) in ddH2O for 30 minutes on ice and washed twice in acetone. We used SDS polyacrylamide gel electrophoresis and Western blotting to quantify the levels of protamines 1 and 2 in sperm nucleus. Total protein (protein assay, Bio-Rad, Hercules, CA, USA) was extracted from sperm nucleus by the NaCl protocol as described above. We quantified the total protein by Qubit 2.0 Fluorometer (Life Technologies) using the Qubit protein assay quantification kit (Q33211, Life Technologies). Subsequently, 20 *μ*g of protein from NaCl pellet was mixed with 5 *μ*L of loading buffer (0.045 M Tris/HCl, 0.8 mM EDTA, 3% SDS 10% glycerol, 5% *β*-mercaptoethanol, and 0.004% bromophenol blue) and loaded into wells. Proteins were separated by dimension on a 12% polyacrylamide gel (*v*/*v*) by standard SDS-PAGE using a Mini protean III System (Bio-Rad, Hercules, CA, USA). A mixture of prestained protein standards was used as marker, with molecular weights ranging from 10 to 250 kDa (Bio-Rad). Electrophoresis was performed for 90 minutes at 130 V at 4°C. Subsequently, proteins were blotted onto polyvinyldenedifluoride (PVDF) membranes by electrophoresis for 120 minutes at 130 mA–300 V (Bio-Rad). After air-drying the membrane, blocking of nonspecific sites was performed with 5% solution (*m*/*v*) skimmed milk powder (Molico®, Nestle Brasil Ltda, São Paulo, Brazil) in PBS for 60 minutes. Membranes were incubated overnight at 4°C with primary antibodies: anti-ovine protamine 1 (mouse monoclonal antibody raised against protamine 1 of ovine origin—Imuny, Campinas, Brazil) or anti-murine protamine 2 (rabbit polyclonal antibody raised against protamine 2 of mouse origin, M-107: sc-30172, Santa Cruz Biotechnology Inc.) diluted 1/500 in PBS-Tween. After three washes every 5 minutes, membranes were incubated with HRP conjugated secondary goat anti-rabbit or goat anti-mouse (Abcam, Cambridge, MA, USA, 1/5000 in PBS, Tween with 1% BSA) for 90 minutes at room temperature and protected from light. Signal intensities and band area quantification were performed after scanning the membranes using Bio-Rad ChemiDoc™ MP (Bio-Rad) and after image analysis using Image Lab 4.0.1 (BioRad). Results were expressed considering the relationship between signal (pixel) and band area. The same protocol was performed in experiment 2 using epididymal sperm.

### 2.2. Experiment 2: Effects of Heat Stress on Epididymal Sperm DNA and Testicular Parenchyma

In this experiment, we evaluated changes in the protamination process caused by heat stress on epididymal sperm and testicular parenchyma. The same animals distributed in the same experimental groups were subjected to a second period of testicular insulation, which lasted 240 hours. This experiment started 60 days after the end of the first experiment. We allowed this interval to ensure that testicular germ cells went through at least one spermatic cycle, minimizing the influence from previous experimental conditions. This was confirmed by routine semen analyses. Animals were assigned to experimental groups as described in experiment 1. Each animal was subjected to two unilateral orchiectomies: the first one was performed 24 hours after the removal of insulation bag (D0) and the second one 30 days after the first orchiectomy (D30). This experimental design considered the ram spermatic cycle (stages I and VIII), in order to include spermatids and spermatozoa in our histology samples. The flow chart representing the experimental design can be viewed in Figure 1.

#### 2.2.1. Obtaining Testicular Fragments

We collected from each testicle fragments weighing 30 mg and measuring approximately 1 cm^3^, in order to execute gene expression and immunohistochemistry analysis. About 500 *μ*L of RNA Later solution (Life Technologies) was added to the fragment to stabilize RNA, as described above. Samples were kept in −80°C.

#### 2.2.2. Epididymal Sperm Collection

After surgery, epididymis was immediately taken to the laboratory and washed in saline solution at 37°C. To collect epididymis semen samples, small incisions were performed with a scalpel blade in the epididymis tail, pressure was applied on its base using hemostats, and sperm were collected with the aid of automatic pipette [32]. Sperm were resuspended in PBS for concentration assessment.

#### 2.2.3. Protamine Assessment in Testicle

*(1) Gene Expression of Protamine 1, Transition Proteins 1 and 2 in Ram Testicle*. RNA isolation, DNA removal, cDNA synthesis, and quantification were performed as described in experiment 1. Due to the likely possibility that each testicular fragment would have different numbers of cells, we used relative q-RT-PCR to determine expression level of protamine 1, transition protein 1, and transition protein 2 gene expression. Primers for protamine 1, transition protein 1, and transition protein 2 were the same as described in experiment 1. Glyceraldehyde 3-phosphate dehydrogenase (GAPDH-foward primer: ATGCCTCCTGCACCACCA, reverse primer: AGTCCCTCCACGATGCAA) and *β*-actin (foward primer CTCTTCCAGCCTTCCTTCCT, reverse primer GGGCAGTGATCTCTTTCTGC) were used as housekeeping genes. Quantitative PCR was performed in triplicates.

*(2) Histology and Immunohistochemistry*. We employed histology analysis and immunohistochemistry in order to identify which cell types are undergoing protamination and apoptosis. Testicular fragments were immediately placed in methanol-Carnoy (methacarn) fixative solution (60% (*v*/*v*) methanol, 30% (*v*/*v*) chloroform, and 10% (*v*/*v*) glacial acetic acid). After 24 hours of fixation in methacarn, fragments were cut down to 0.5 cm^3^ and immersed in 95% ethanol (*v*/*v*) prior to processing and embedding in paraffin. Sections of 5 *μ*m were stained with hematoxylin and eosin to access histological lesions. Sections were placed on silanized slides and submitted to immunohistochemistry for PRM1 and PRM2, BAX, and BCL2. Blocking of endogenous peroxidase was performed with 5% hydrogen peroxide solution (*v*/*v*) in methanol (1 : 1) for 30 minutes, and antigen retrieval was performed in a water bath with 10 mM sodium citrate buffer pH 6.0 at 96°C for 20 minutes. Blocking of nonspecific sites with 5% solution (*m*/*v*) skimmed milk powder in PBS was carried at room temperature for 60 minutes. Mouse monoclonal antibody raised against protamine 1 of ovine origin (Imuny, Campinas, Brazil) or rabbit polyclonal antibody raised against protamine 2 of mouse origin (sc-30172, Santa Cruz Biotechnology Inc., CA, USA) was employed to identify cellular types undergoing sperm protamination. Rabbit polyclonal anti-murine BAX (sc-526, Santa Cruz Biotechnology) or rabbit polyclonal anti-human BCL2 (sc-492, Santa Cruz Biotechnology) antibodies were used to identify cellular types undergoing apoptosis mediated by action of reactive oxygen species. Fragments of intestine and mammary gland were used as positive control. Antibodies were diluted 1 : 100 in PBS and applied on slides in a humid chamber at 4°C for 16–18 h. We used homologous nonimmune serum as a negative control. After incubation and washing in PBS, slides were incubated for 30 min with secondary anti-mouse and anti-rabbit antibodies in a Tris-HCl buffer (Link Advance HRP™, Dako-Agilent Technologies, Santa Clara, USA). After further washing in PBS, slides were incubated for 30 minutes with peroxidase-polymerized antibody in Tris-HCl buffer (Enzyme Advance HRP, Dako-Agilent Technologies). Later, we proceeded to incubation with chromogen (Vector NovaRed Substrate Kit, SK4800, Vector Laboratories, CA, USA) for 2 minutes or until slides presented a light pink color. Hematoxylin counter staining was used only in slides incubated with anti-BAX or anti-BCL2 primary antibodies. Evaluation of histological and immunohistochemistry slides was performed in an optical light microscope (Nikon Eclipse Ni) coupled to a digital camera (Nikon DS-Ri1), and images were obtained by NIS Elements® program. Cell types immunolabeled for PRM1, PRM2, BAX, and BCL2 in the ram seminiferous tubules were identified based on morphology.

## 3. Statistical Analysis

Statistical Analysis System 9.3 (SAS Institute, Cary, NC) was used to analyze the dependent variables. All data were tested for normality of residues and homogeneity of variances. Variables that did not comply with these statistical premises were transformed. In experiment 1, the MIXED procedure was used for analysis of variance with repeated measures over time. Comparisons of means were performed using *least square means* (LS means) for different dependent variables and for each condition of the statistical model (treatment, week, and treatment versus week). Considering nonparametric data, we used nonparametric analysis of variance (Kruskal-Wallis, through NPAR1WAY procedure) and pairwise comparison of means (Wilcoxon). Parametric results are presented as mean ± SEM. Nonparametric results are presented as median (low quartile, high quartile). In experiment 2, analysis of variance was carried using GLM procedure considering the 2 × 2 factorial design. Factors considered treatment effect (treated group versus control group) and effect of time represented by first (D0) and second (D30) unilateral orchiectomies, with subsequent comparison of means by the least significant difference (LSD) method. A 5% significance level was used to reject the null hypothesis. The qPCR data in testicular samples were analyzed by MIXED procedure, as previously described [33].

## 4. Results

### 4.1. Experiment 1: Effects of Heat Stress on Ejaculated Sperm DNA

**Figure 2:**
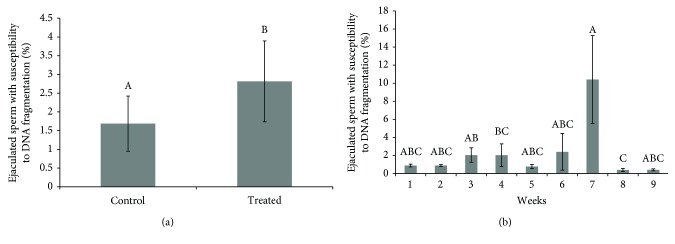
(a) Percentage of sperm with fragmentation, in the control (*n* = 54) versus treated (*n* = 54) groups. (b) Percentage of sperm with fragmentation in different weeks (*n* = 108). The results are presented as means ± SEM. Different superscript letters in each bar represent significant differences (*p* < 0.05).

The aim of this study was to evaluate sperm DNA fragmentation, chromatin structure, protamine deficiency in sperm chromatin, gene expression of protamines, and transition proteins in ejaculated sperm of rams submitted to heat stress by testicular insulation. Treated group (2.81% ± 1.08) exhibited an increase (*p* < 0.047) in the percentage of sperm cells that were positive for acridine orange versus the control group (1.68 ± 0.73%) (Figure 2(a)). An effect of time can also be observed, as there was an increase in the percentage of sperm cells that were positive for acridine orange in the seventh experimental week; however, other relevant differences in the remaining studied weeks were not observed (*p* > 0.05) (Figure 2(b)).

**Figure 3:**
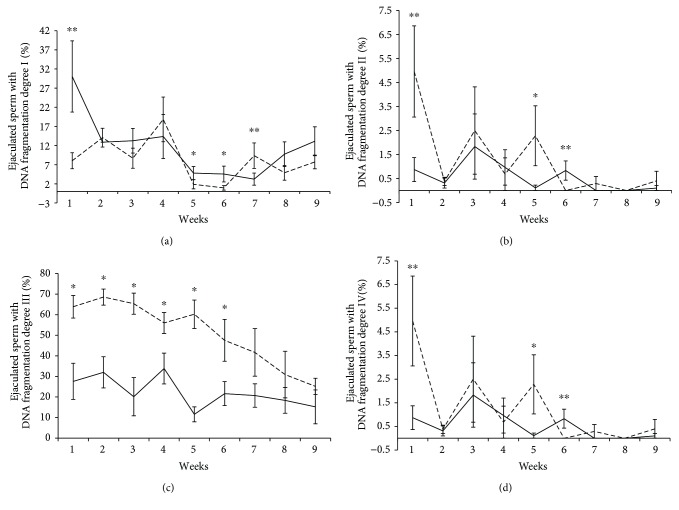
Comet assay results from the control (*n* = 54) versus treated (*n* = 54) groups for different grades of chromatin fragmentation (I, II, III, and IV) over nine experimental weeks. (a) Percentage of grade I sperm from the control versus treated groups over nine experimental weeks. (b) Percentage of grade II sperm from the control versus treated groups over nine experimental weeks. (c) Percentage of grade III sperm from the control versus treated groups over nine experimental weeks. (d) Percentage of grade IV sperm from the control versus treated groups over nine experimental weeks. The results are presented as mean ± SEM. Different superscript letters in each bar represent significant differences (*p* < 0.05). Control group = solid line. Treated group = dashed line ^∗^*p* < 0.05, ^∗∗^0.05 < *p* < 0.10.

Quantitative analysis of sperm DNA damage by alkaline Comet technique revealed that the treated group presented a lower number of grade II sperm DNA fragmentation (Figure 3(b)) and a higher number of grade III sperm DNA fragmentation (Figure 3(c)), when compared to the control group. From the sixth experimental week onwards, these differences were no longer apparent. The treated group presented a small percentage of grade I sperm since the first week after testicular insulation (8.03% ± 2.09) when compared to the control group (30.05% ± 9.29), indicating an acute response to testicular insulation (Figure 3(a)). In the treated group, we observed a trend (*p* = 0.07) for an increase in the percentage of grade IV sperm (Figure 3(d); 4.96% ± 1.90) when compared to the control group (0.87% ± 0.50). The mean values, standard errors of averages, and probability for each degree of sperm DNA fragmentation, in each experimental week, are available in Table 1.

No differences (*p* > 0.05) were observed between the treated group (0.185% (0.1; 0.37)) and control (0.27% (0.12; 0.45)) in the percentage of ejaculated sperm enclosing protamine deficiency as assessed by CMA3 flow cytometry. An effect of time was not observed for this variable. PRM1, TNP1, and TNP2 gene expression in ejaculated sperm were verified by qPCR. The treated group presented a decrease in the number of mRNA TNP1 copies (*p* = 0.042) when compared to the control group (4.09 ± 1.02 versus 17.19 ± 4.84, resp.). No differences in gene expression of PRM1 (*p* = 0.63) and TNP2 (*p* = 0.55) were observed between the groups (Table 2). An effect of time was not observed for all studied genes.

PRM1 and PRM2 protein expression in ejaculated sperm were verified by Western blotting. No differences in protein expression of PRM 1 (*p* = 0.23) were observed (control group = 559736.67 ± 181961.46; treated group = 936606.22 ± 257729.83). Furthermore, no differences were observed between the groups over the experimental weeks (*p* = 0.094). No differences in protein expression of PRM 2 (*p* = 0.43) were observed (control group = 1079265.56 ± 244383.62; treated group = 849526.22 ± 170714.51), and no differences were observed over the experimental weeks (*p* = 0.62).

### 4.2. Experiment 2: Effects of Heat Stress on Epididymal Sperm DNA and Testicular Parenchyma

Surprisingly, no differences were observed (*p* = 0.60) after evaluation of sperm DNA susceptibility to denaturation by acridine orange staining between the treated and control groups in epididymal sperm (1.40% ± 0.41 versus 1 02% ± 0.23). No differences were observed (*p* = 0.14) in insulation effect when comparing D0 to D30 (0.68% ± 0.48 and 1.62 ± 1.37%, resp.).

Regarding gene expression on epididymal sperm, no differences were observed in PRM1, TNP1, and TNP2 gene expression between the control and treated groups (Table 3). Regarding the effect of time, considering D0 and D30, PRM1 (*p* < 0.0001) showed higher expression in D30 when compared to D0 (10.745 ± 2.87 versus 0.92 ± 0.128) (Table 3). No differences were observed in gene expression of TNP1 (*p* = 0.12) and TNP2 (*p* = 0.24) when the effect of time was studied (Table 4).

**Figure 4:**
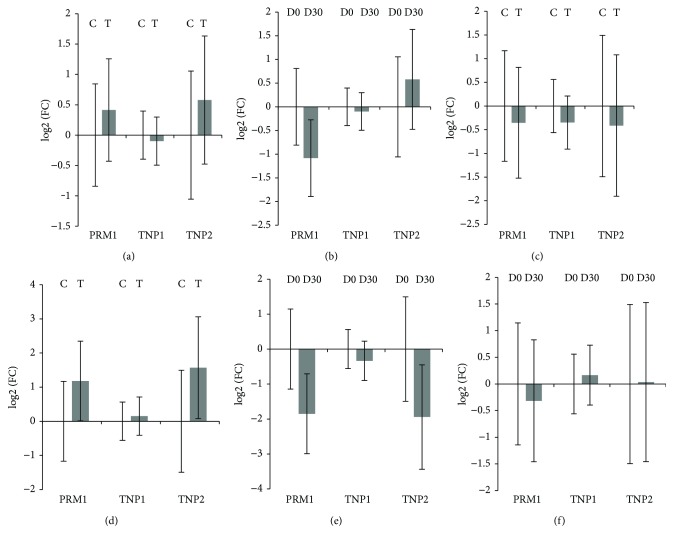
Relative gene expression of PRM1, TNP1, and TNP2. (a) Comparison between the control (*n* = 108) and treated (*n* = 108) groups. (b) Comparison between D0 (*n* = 108) and D30 (*n* = 108) of the spermatic cycle. (c) Comparison between the control (*n* = 54) and treated (*n* = 54) groups at D0 of the spermatic cycle. (d) Comparison between the control (*n* = 54) and treated (*n* = 54) groups at D30 of the spermatic cycle. (e) Comparison between D0 (*n* = 54) and D30 (*n* = 54) of the spermatic cycle within the control group. (f) Comparison between D0 (*n* = 54) and D30 (*n* = 54) of the spermatic cycle within the treated group. Results are presented as mean ± SEM. Amounts expressed in log2 (FC). FC = fold change. C = control group. T = treated group.

The expression of target genes (PRM1, TNP1, and TNP2) in testicles was not different when we evaluated the control or treated groups and effect of times (D0 and D30) (*p* > 0.05) (Figure 4 and Table 5).

PRM1 and PRM2 protein expression in epididymal sperm were verified by Western blotting. No differences in protein expression of PRM 1 at D0 (*p* = 0.2805) were observed (control group = 709134.60 ± 138887.29; treated group = 516909.33 ± 100372.69). Furthermore, no differences were observed between the groups (control group = 440066.40 ± 147984.87; treated group = 533607.20 ± 139959.44) at D30 of sperm protamination (*p* = 0.65). The quantification of PRM2 at D0 was not performed due to technical problems. No differences in protein expression of PRM 2 at D30 (*p* = 0.98) were observed (control group = 699536.0 ± 134559.64; treated group = 704750.4 ± 202385.65).

In order to assess protamination and apoptosis pathways induced by ROS, we performed histological analysis and immunohistochemistry against protamine 1, protamine 2, BAX, and BCL2 proteins in seminiferous tubules submitted to heat stress by testicular insulation. We considered the effect of heat stress (control versus treated) and the effect of time (D0 versus D30).

**Figure 5:**
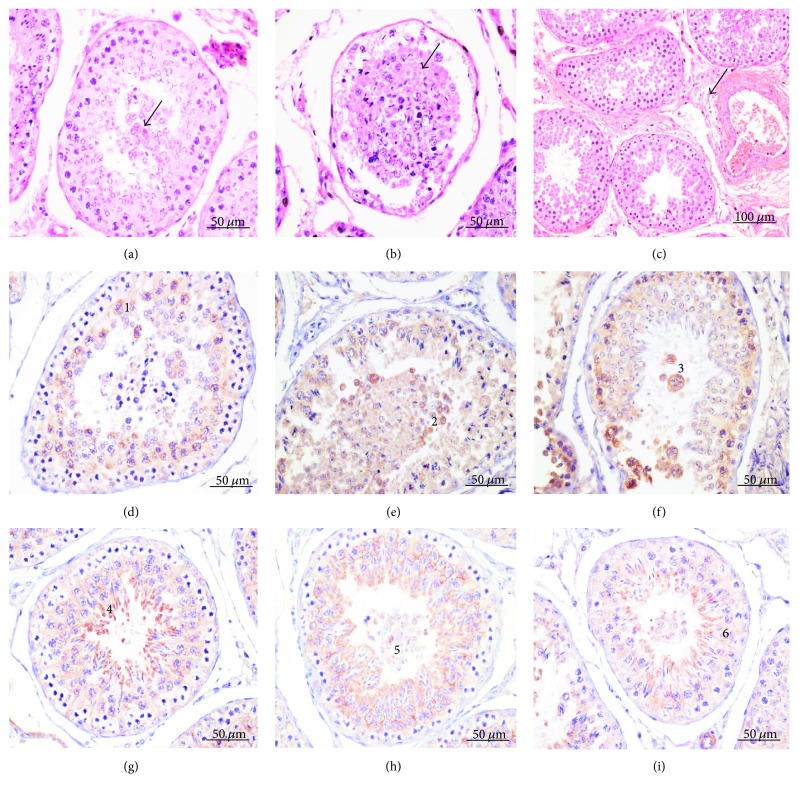
Optical microscopy of ram testis after testicular insulation stained with hematoxylin and eosin and representative images of immunohistochemical localization of BAX (rabbit polyclonal anti-murine) and BCL2 (rabbit polyclonal anti-human) in ram seminiferous tubules. (a) Cell desquamation with giant cells in the tubular lumen (arrow), 400x magnification. (b) Seminiferous tubules showing flaking germ cells into the tubular lumen (arrow), 400x magnification. (c) Mild multifocal interstitial fibrosis (arrow), 200x magnification. (d) Intracytoplasmic labeling of BAX in spermatocytes I (1), 400x magnification. (e) Intracytoplasmic labeling of BAX in squamous cells present in the lumen (2), 400x magnification. (f) Intracytoplasmic labeling of BAX in binucleated squamous cells in the lumen, 400x magnification. (g) Intracytoplasmic labeling of BCL2 in elongated spermatids (4), 400x magnification. (i) Mild intracytoplasmic labeling of BCL2 in cells present in the lumen (5), 400x magnification. (i) Less intense intracytoplasmic labeling of BCL2 in spermatocytes I than spermatids (6), 400x magnification.

The treated group showed some histological features of initial testicular degeneration. Mild and multifocal vacuolization of Sertoli cells was observed, with a small to moderate number of desquamated cells containing three or more nuclei (giant cells) inside the seminiferous tubules (Figure 5(a)). In addition, there was desquamation of germ cells (Figure 5(b)) and mild multifocal interstitial fibrosis (Figure 5(c)). Histological lesions in the control group were not observed. When examining differences between spermatic cycle stages in the seminiferous tubules, a higher number of secondary spermatocytes in D30 were observed when compared to D0. There was a decrease in the number of spermatogonia in D30. There were lower number of desquamated cells within seminiferous tubules and a small proliferation of less differentiated cells, as well as spermatogonia, in D30 compared to D0, suggesting regeneration.

**Figure 6:**
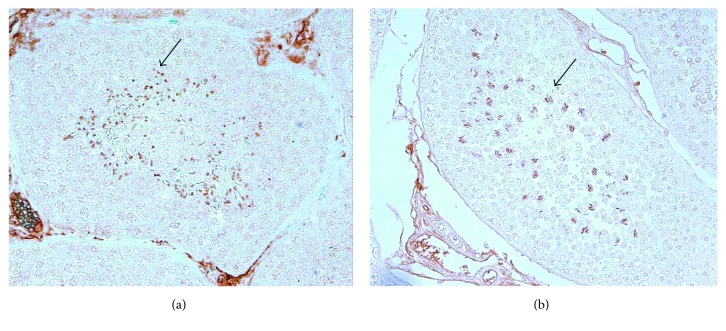
Representative images of immunohistochemical localization of PRM1 (mouse monoclonal antibody raised against protamine 1 of ovine origin) (a) and PRM2 (rabbit polyclonal antibody raised against protamine 2 of mouse origin) (b) in ram seminiferous tubules by optical microscopy (arrows), 400x magnification.

We used immunohistochemistry to determine whether oxidative stress alters protein expression of protamines. PRM1 and PRM2 were detected in differentiated cells, like spermatids and sperm (Figures 6(a) and 6(b)). No differences were observed between control and treated groups, or during the examination of the effect of time (D0 and D30).

To determine whether oxidative stress induces changes in the activation of apoptotic pathways, we investigated the proapoptotic protein BAX. In the control group, a diffuse granular intracytoplasmic labeling in both primary and secondary spermatocytes in D0 was observed (Figure 5(d)), but predominant in primary spermatocytes. Similarly, the treated group presented BAX labeling within the primary spermatocyte cytoplasm, with the distinction that all desquamated and binucleated cells were labeled (Figures 5(e) and 5(f)) within the seminiferous tubule lumina. In addition, no differences in BAX imunolabeling between the control and treated groups at D30 were observed. The antiapoptotic protein BCL2 was also investigated. No differences were observed between the control and treated groups, or during assessment of the effect of time. In general, an intense intracytoplasmic labeling for BCL2 was observed in spermatids (Figure 5(g)). A less intense labeling for BCL2 was observed within the cytoplasm of desquamated cells present within seminiferous tubule lumina (Figure 5(h)), such as primary spermatocytes (Figure 5(i)).

## 5. Discussion

Sperm that comprise chromatin damage are still able to fertilize an egg; however, this may be critical in the etiology of infertility, particularly in idiopathic cases [1]. Heat stress is one of the causes of augmented sperm DNA fragmentation by increased oxidative stress, failures in compaction of chromatin, and apoptosis in the mouse [34]. In this study, we assessed effect of heat stress on semen and testicles of rams, focusing on sperm DNA fragmentation, susceptibility to sperm chromatin fragmentation, protamine deficiency, gene or protein expression of protamines and transition proteins, and the presence of pro- and antiapoptotic proteins in spermatocytes and spermatids.

When analyzing sperm from the ejaculate, we observed an increase in the percentage of sperm susceptible to DNA acid denaturation and in the percentage of grade III sperm after Comet assay in the treated group. Our previous published study [29] proposed that heat stress promoted by testicular insulation causes oxidative stress. Therefore, these new results suggest that oxidative stress promotes DNA fragmentation. This assertion is substantiated by the increase in the percentage of sperm with evident chromatin damage (grade III) observed in the sixth experimental week. The population of sperm grade II decreased in the treated group as cells evolved to grade III, implying that the increased fragmentation observed in the treated group would occur in sperm with previous DNA damage and presumable chromatin alteration. Assessment of sperm DNA susceptibility to denaturation promotes chromatin denaturation where DNA damage already exists [35]. Whereas alkaline comet is a technique used to quantify the grade of DNA fragmentation, it is complementary to analysis of sperm DNA susceptibility to denaturation. This relationship between sperm DNA susceptibility to denaturation and DNA fragmentation was also observed in cattle [36, 37]. Sperm with higher susceptibility to DNA fragmentation would suffer chromatin damage quickly under oxidative stress condition [38]. This was already reported in human patients with varicocele, in which increased DNA damage was associated with oxidative stress induced by heat stress [38].

The absence of differences between groups when considering epididymal sperm DNA susceptibility to damage after the second insulation was unexpected. A plausible explanation is that sperm cells that were more susceptible to heat stress were eliminated in the first insulation. This mechanism would be similar to what occurs in the hypoxia-reperfusion syndrome [39] after organ transplantation. Oxidative damage may occur due to the increased oxygen supply during tissue reperfusion after hypoxia, generating a condition of oxidative stress due to the increased oxygen supply [40–42]. This leads to an increase in apoptosis that can be beneficial in the long term, as a negative correlation was observed between the degree of apoptosis at the time of transplantation and the concentration of serum creatinine six months after renal transplants [43]. Similarly, the testis works in hypoxia conditions, since it is poorly vascularized [44]. In the case of an increase in testicular temperature, metabolic activity increases without efficient vasodilatation, leading to hypoxia [44, 45]. When testis temperature returns to normal, reoxygenation occurs, similarly to what happens after organ transplantation. Thus, the higher amount of DNA fragmentation observed after heat stress could reflect an increase in apoptosis during the first experiment. Animals with increased sperm susceptibility to DNA damage would have these cells more efficiently eliminated. This fact is supported by the decrease in the number of spermatogonia observed in D30. This finding may be related to increased cell differentiation due to tissue recovery after heat stress. Thus, in a possible subsequent stress, these cells would be less prone to DNA damage.

The decrease in TNP1 mRNA copies in ejaculated sperm from the treated group could reflect higher consumption of mRNA due to a possible need for higher DNA compaction in this group. Gene expression in mature spermatozoa progressively ceases during spermiogenesis to allow compaction of the genome, replacing histones by the protamines [14, 15]. In fact, Feugang et al. [46] verified that mature sperm might exhibit transient translational activity to replace protamines that could be defective. No difference between the control and treated groups was observed when we assessed protamination per se using the CMA3 techniques in ejaculated sperm, showing that the final outcome is unchanged in spite of the change in gene expression of TNP1.

Gene expression analysis in ram epididymal sperm revealed that only PRM1 showed a higher number of copies in D30. This increase could be an effect from the orchiectomy. The removal of the left testicle may have stimulated an impairment of spermatogenesis in the right testicle, and consequently lack of mRNA consumption. Interestingly, when we assessed testicular material subjected to the same treatment, no differences were observed. When using testicular material for RNA extraction, all cell types were lysed. Since we identified PRM1 proteins preferably in spermatids, isolation and analysis of spermatid mRNA would provide a clearer picture of changes in PRM1 mRNA transcription and consumption during spermatogenesis.

Considering the activation of apoptotic pathways, we observed that BAX was detected preferably in spermatocytes, whereas BCL2 was detected in spermatids. Spermatocytes showed increased marking by BAX, as already described in rodents [47], monkeys [48], and human [49]; however, heat stress did not increase cell types undergoing apoptosis. As expected, desquamated and binucleated cells were BAX positive, indicating that these cells underwent apoptosis. On the other hand, spermatids showed an increase on antiapoptotic mechanism reflected by higher BCL2 staining, which is consistent with the idea that mature sperm should maintain higher levels of antiapoptotic proteins [50]. These high BCL2 levels may be related to spermatozoa that are hallmarked for apoptosis but escape from it, a process normally called “abortive apoptosis” [51]. To our knowledge, this is the first description of cell types involved in the apoptotic pathways mediated by ROS in ram testicles subjected to heat-induced oxidative stress.

In conclusion, heat stress increases sperm DNA fragmentation and chromatin alterations without significant changes in the protamination process or in cell types undergoing apoptosis. Unexpectedly, when animals were submitted to a second heat stress, less DNA fragmentation was observed, even after a resting period. This suggests that susceptible cells were affected and eliminated from the testicular environment. Novel experiments could be performed to verify if previous exposure to heat stress leads to a more resistant testicular structure, resulting in less overall damage to sperm chromatin.

## Figures and Tables

**Table 1:** Means (%) ± SEM and *p* value of ram sperm presenting different degrees of DNA fragmentation. Grade I (few damages), grade II (light damages), grade III (evident damage), and grade IV (intense damage) in different experimental weeks, in the control and treated groups.

Grade	Week	Control	Treated	*p*
I	1	30.05 ± 9.29	8.03% ± 2.09	*0.06*
	2	12.97% ± 4.33	14.06 ± 2.45	0.19
	3	13.25% ± 3.23	8.67 ± 2.62	0.19
	4	14.36% ± 5.76	18.83 ± 5.83	0.24
	5	4.80% ± 1.71	1.87 ± 1.20	0.54
	6	4.5% ± 2.07	0.94% ± 0.60	**0.04**
	7	3.23% ± 1.59	9.35% ± 3.33	*0.06*
	8	9.75% ± 3.22	4.89% ± 1.94	0.16
	9	13.12% ± 3.78	7.71% ± 1.79	015

II	1	41.46% ± 7.35	23.10% ± 5.56	*0.06*
	2	54.80% ± 6.14	16.98% ± 3.38	**0.001**
	3	64.76% ± 10.25	23.47% ± 6.93	**0.013**
	4	50.83% ± 12.83	24.49% 6.10	**0.02**
	5	83.49% ± 3.84	35.58% ± 5.58	**0.001**
	6	72.95% ± 4.90	51.52% ± 10.14	0.12
	7	76.06% ± 5.96	48.66% ± 11.25	*0.06*
	8	71.90% ± 4.17	64.19% ± 10.43	0.46
	9	71.56% ± 4.36	66.68% ± 4.90	0.34

III	1	27.61% ± 8.78	64.85% 5.50	**0.002**
	2	32.00% ± 7.58	68.57% ± 3.91	**0.001**
	3	20.14% ± 9.26	65.35 ± 5.12	**0.007**
	4	33.84% ± 7.49	55.99% ± 5.07	**0.02**
	5	11.58% ± 3.63	60.25% ± 6.90	**0.0011**
	6	21.65% ± 5.80	47.52% ± 10.18	**0.03**
	7	20.70% ± 5.70	41.68% ± 11.57	0.12
	8	18.34% ± 6.25	30.90% ± 11.30	0.34
	9	15.22% ± 8.24	25.19% ± 3.93	**0.04**

IV	1	0.87% ± 0.50	4.96% ± 1.90	*0.07*
	2	0.31% ± 0.21	0.38% ± 0.17	0.42
	3	1.83% ± 1.36	2.50% ± 1.82	0.43
	4	0.96% ± 0.74	0.68% ± 0.68	0.21
	5	0.11% ± 0.11	2.28% ± 1.25	**0.02**
	6	0.83% ± 0.40	0	*0.09*
	7	0	0.29% ± 0.29	0.50
	8	0	0	1
	9	0.10% ± 0.10	0.40% ± 0.40	0.50

**Table 2:** Gene expression of PRM1, TNP1, and TNP2 in ejaculated ram sperm presented as number of copies considering treatment effect. Mean ± SEM and *p* value.

Target genes	Control group	Treated group	*p*
PRM1	31.11 ± 13.69	19.59 ± 5.85	0.63
TNP1	17.19 ± 4.84	4.09 ± 1.02	**0.042**
TNP2	1.97 ± 0.79	1.01 ± 0.14	0.55

**Table 3:** Gene expression of PRM1, TNP1, and TNP2 in epididymal ram sperm presented as number of copies considering treatment effect. Mean ± SEM and *p* value.

Target genes	Control group	Treated group	*p*
PRM1	6.73 ± 3.01	8.21 ± 3.81	0.86
TNP1	2.76 ± 0.88	2.14 ± 0.65	0.64
TNP2	0.35 ± 0.03	0.56 ± 0.19	0.34

**Table 4:** Gene expression of PRM1, TNP1, and TNP2 in epididymal ram sperm presented as number of copies considering effect of time (D0 versus D30). Mean ± SEM and *p* value.

Target genes	D0	D30	*p*
PRM1 mRNA copies	0.92 ± 0.12	10.74 ± 2.87	**<0.0001**
TNP1 mRNA copies	2.81 ± 0.61	0.94 ± 0.18	0.12
TNP2 mRNA copies	0.39 ± 0.08	0.72 ± 0.35	0.24

**Table 5:** Probability values (*p*) for PRM1, TNP1, and TNP2 gene expression in ram testis submitted to heat stress considering the different effects analyzed.

Effects	PRM1	TNP1	TNP2
Treatment (control versus treated)	0.6347	0.8066	0.5903
Protamination (D0 versus D30)	0.2099	0.8324	0.3767
D0: control versus treated	0.7649	0.5405	0.7851
D30: control versus treated	0.3243	0.7884	0.3061
Control group: D0 versus D30	0.1365	0.5559	0.2075
Treated group: D0 versus D30	0.7880	0.7705	0.9811
